# Supplementary material for: The psychosocial burden of women seeking treatment for breast and cervical cancers in Ghana’s major cancer hospitals
Source: PLoS One. 2023 Aug 22;18(8):e0289055. doi: 10.1371/journal.pone.0289055 (PMC10443842; doi:10.1371/journal.pone.0289055)
Supplement: Attachment — Submitted filename: PONE-D-23-01781_BM.pdf [file pone.0289055.s001.pdf]

PLOS ONE

The psychosocial burden of women seeking treatment for Breast and Cervical Cancers in Ghana's major Cancer Hospitals

--Manuscript Draft--

**Manuscript Number:**	PONE-D-23-01781
**Article Type:**	Research Article
**Full Title:**	The psychosocial burden of women seeking treatment for Breast and Cervical Cancers in Ghana's major Cancer Hospitals
**Short Title:**	Psychosocial burden of cancer treatment in Ghana
**Corresponding Author:**	Edward Nketiah-Amponsah, PhD<br>University of Ghana<br>Accra, GHANA
**Keywords:**	Psychosocial burden, intangible cost, breast cancer, cervical cancer, women, cancer patients, cancer treatment, mixed method study, Ghana.
**Abstract:**	Background: Breast and cervical cancers remain the most common cancers and the leading cause of cancer deaths in Ghana. Apart from the huge economic cost cancer treatment poses to patients and their families, they also bear high intangible or psychosocial burdens from the treatment.<br>Methods: A convergent mixed method approach was used to ascertain the psychosocial experiences or intangible cost of breast and cervical cancer patients who sought treatment in three leading cancer hospitals in Ghana. Primary data were collected using a questionnaire and an interview guide from 298 breast and cervical cancer patients, their family members and health care providers at the Korle-Bu and Komfo Anokye Teaching Hospitals as well as the Sweden Ghana Medical Centre. Qualitative analysis was done using thematic content analysis while quantitative analysis was done using logistic regression.<br>Results: The findings of the study showed that patients not only battled with psychological issues such as anxiety, pain, stigma, fear of death and loss of spouses, but also struggled with physical and social restrictions. Patients with low educational level and income status, retired or unemployed, and/or had larger household size suffered more intangible cost or psychosocial burden.<br>Conclusion: There is an urgent need for psychosocial support for breast and cervical cancer patients studied, to enhance their quality of life. While educational campaigns on mainstream and social media have contributed to increased awareness on breast and cervical cancers in Ghana, similar intensified educational campaigns are needed in Ghana, to demystify the stigma surrounding cancers.
**Order of Authors:**	Patience Nana Amoa Gyamenah
	Adobea Yaa Owusu
	Joseph Ransford Oppong
	Kingsley E Amegah
	Edward Nketiah-Amponsah, PhD
**Additional Information:**	
**Question**	**Response**
**Financial Disclosure**<br><br>Enter a financial disclosure statement that describes the sources of funding for the work included in this submission. Review the submission guidelines for detailed requirements. View published research articles from *PLOS ONE* for specific	Not applicable

examples.<br><br>This statement is required for submission and **will appear in the published article** if the submission is accepted. Please make sure it is accurate.<br><br>**Unfunded studies**<br>Enter: *The author(s) received no specific funding for this work.*<br><br>**Funded studies**<br>Enter a statement with the following details:<br>• Initials of the authors who received each award<br>• Grant numbers awarded to each author<br>• The full name of each funder<br>• URL of each funder website<br>• Did the sponsors or funders play any role in the study design, data collection and analysis, decision to publish, or preparation of the manuscript?<br>• **NO** - Include this sentence at the end of your statement: *The funders had no role in study design, data collection and analysis, decision to publish, or preparation of the manuscript.*<br>• **YES** - Specify the role(s) played.<br><br>* typeset	
**Competing Interests**<br><br>Use the instructions below to enter a competing interest statement for this submission. On behalf of all authors, disclose any competing interests that could be perceived to bias this work—acknowledging all financial support and any other relevant financial or non-financial competing interests.<br><br>This statement is required for submission and **will appear in the published article** if the submission is accepted. Please make sure it is accurate and that any funding sources listed in your Funding Information later in the submission form are also declared in your Financial Disclosure statement.	The authors have declared that no competing interests exist.

View published research articles from *PLOS ONE* for specific examples.<br><br>**NO authors have competing interests**<br><br>Enter: *The authors have declared that no competing interests exist.*<br><br>**Authors with competing interests**<br><br>Enter competing interest details beginning with this statement:<br><br>*I have read the journal's policy and the authors of this manuscript have the following competing interests: [insert competing interests here]*<br><br>* typeset	
**Ethics Statement**<br><br>Enter an ethics statement for this submission. This statement is required if the study involved:<br><br>• Human participants<br>• Human specimens or tissue<br>• Vertebrate animals or cephalopods<br>• Vertebrate embryos or tissues<br>• Field research<br><br>Write "N/A" if the submission does not require an ethics statement.<br><br>General guidance is provided below. Consult the submission guidelines for detailed instructions. **Make sure that all information entered here is included in the Methods section of the manuscript.**	Ethical clearance was obtained from the Ethics Committee for Humanities of the University of Ghana (Ref. No ECH011/19-20), the Korle-Bu Teaching Hospital Ethical Review Board (Ref. No KBTH/MD/G8/20), and the Komfo Anokye Teaching Hospital Review Board (Ref. No KATH-IRB/AP/023/20). Informed written consent was obtained from all study participants before interviews were conducted. Permission was also obtained from research participants to record interviews for the purposes of data analysis and for writing academic-related reports.

**Format for specific study types**<br><br>**Human Subject Research (involving human participants and/or tissue)**<br>• Give the name of the institutional review board or ethics committee that approved the study<br>• Include the approval number and/or a statement indicating approval of this research<br>• Indicate the form of consent obtained (written/oral) or the reason that consent was not obtained (e.g. the data were analyzed anonymously)<br><br>**Animal Research (involving vertebrate animals, embryos or tissues)**<br>• Provide the name of the Institutional Animal Care and Use Committee (IACUC) or other relevant ethics board that reviewed the study protocol, and indicate whether they approved this research or granted a formal waiver of ethical approval<br>• Include an approval number if one was obtained<br>• If the study involved *non-human primates*, add *additional details* about animal welfare and steps taken to ameliorate suffering<br>• If anesthesia, euthanasia, or any kind of animal sacrifice is part of the study, include briefly which substances and/or methods were applied<br><br>**Field Research**<br><br>Include the following details if this study involves the collection of plant, animal, or other materials from a natural setting:<br>• Field permit number<br>• Name of the institution or relevant body that granted permission	
**Data Availability**<br><br>Authors are required to make all data underlying the findings described fully available, without restriction, and from the time of publication. PLOS allows rare exceptions to address legal and ethical concerns. See the PLOS Data Policy and FAQ for detailed information.	No - some restrictions will apply

A Data Availability Statement describing where the data can be found is required at submission. Your answers to this question constitute the Data Availability Statement and **will be published in the article**, if accepted.<br><br>**Important:** Stating 'data available on request from the author' is not sufficient. If your data are only available upon request, select 'No' for the first question and explain your exceptional situation in the text box.<br><br>Do the authors confirm that all data underlying the findings described in their manuscript are fully available without restriction?	
**Describe where the data may be found in full sentences. If you are copying our sample text, replace any instances of XXX with the appropriate details.**<br><br>• If the data are **held or will be held in a public repository**, include URLs, accession numbers or DOIs. If this information will only be available after acceptance, indicate this by ticking the box below. For example: *All XXX files are available from the XXX database (accession number(s) XXX, XXX.).*<br>• If the data are all contained **within the manuscript and/or Supporting Information files**, enter the following: *All relevant data are within the manuscript and its Supporting Information files.*<br>• If neither of these applies but you are able to provide **details of access elsewhere**, with or without limitations, please do so. For example:<br><br>*Data cannot be shared publicly because of [XXX]. Data are available from the XXX Institutional Data Access / Ethics Committee (contact via XXX) for researchers who meet the criteria for access to confidential data.*<br><br>*The data underlying the results presented in the study are available from (include the name of the third party*	The data for this paper are available upon reasonable request from Dr. Patience Nana Amoa Gyamenah, upon a formal request. There are ethical and confidential concerns about breast cancer within the Ghanaian cultural setting. The patients were promised that their responses will not be shared with the public, unless it becomes necessary to share it with persons who may request to use it privately—or to Editors of publishing outlets, when some of them showed concerns during the interviews about keeping the data confidential.

and contact information or URL).<br>• This text is appropriate if the data are owned by a third party and authors do not have permission to share the data.<br><br>* typeset	
Additional data availability information:	Tick here if the URLs/accession numbers/DOIs will be available only after acceptance of the manuscript for publication so that we can ensure their inclusion before publication.

Manuscript

Click here to access/download;Manuscript;Manuscript_Psychosocial

The Psychosocial Burden of Women Seeking Treatment for Breast and Cervical Cancers in Ghana's Major Cancer Hospitals

Patience Nana Amoa Gyamenah[1],

Adobea Yaa Owusu[2], ORCID: 0000-0003-2223-7896

Joseph Ransford Oppong[3],

Kingsley E Amegah[4],

*Edward Nketiah-Amponsah[5], ORCID: 0000-0002-9754-1983

[1,2]Institute of Statistical, Social and Economic Research. University of Ghana, Legon. Accra, Ghana.

[3]Department of Geography, University of North Texas. Denton, Texas, United States of America.

[4]University of Cape Coast, School of Economics, Department of Data Science and Economic Policy.

[*5]Department of Economics, University of Ghana, Legon. Accra, Ghana. enamponsah@ug.edu.gh

Abstract

Background: Breast and cervical cancers remain the most common cancers and the leading cause of cancer deaths in Ghana. Apart from the huge economic cost cancer treatment poses to patients and their families, they also bear high intangible or psychosocial burdens from the treatment.

Methods: A convergent mixed method approach was used to ascertain the psychosocial experiences or intangible cost of breast and cervical cancer patients who sought treatment in three leading cancer hospitals in Ghana. Primary data were collected using a questionnaire and an interview guide from 298 breast and cervical cancer patients, their family members and health care providers at the Korle-Bu and Komfo Anokye Teaching Hospitals as well as the Sweden Ghana Medical Centre. Qualitative analysis was done using thematic content analysis while quantitative analysis was done using logistic regression.

Results: The findings of the study showed that patients ~~not only~~ battled with psychological issues such as anxiety, pain, stigma, fear of death and loss of spouses, but also struggled with physical and social restrictions. Patients with low educational level and income status, retired or unemployed, and/or had larger household size suffered ~~more intangible cost or~~ psychosocial burden.

Conclusion: There is an urgent need for psychosocial support for breast and cervical cancer patients studied, to enhance their quality of life. While educational campaigns on mainstream and social media have contributed to increased awareness on breast and cervical cancers in Ghana, similar intensified educational campaigns are needed in Ghana, to demystify the stigma surrounding cancers.

Key Words: Psychosocial burden, intangible cost, breast cancer, cervical cancer, women, cancer patients, cancer treatment, mixed method study, Ghana.

1.0 Introduction

Good health is directly linked with increased productivity, hence the emphasis on good health and well-being for all by the Sustainable Development Goal three (SDG 3). Unfortunately, the global rising burden of non-communicable diseases (NCDs) such as cancer, stroke, hypertension, and cardiovascular diseases, especially among women, has become a considerable health and development issue. These pose a challenge to the achievement of SDG goals 3 and 5 [1].

About 19.3 million new cancer cases and 10 million cancer deaths were recorded globally in 2020 [2]. Female breast cancer was the most commonly diagnosed cancer with an incidence of 2.26 million cases, closely followed by lung cancer with an incidence of 2.21 million cases [3]. The most common cause of cancer deaths worldwide was lung cancer followed by liver and stomach cancers [2]. Breast cancer was the leading cause of cancer deaths in twelve regions of the world while cervical cancer was the leading cause of death in three regions in Sub Saharan Africa including Eastern Africa [3]. It is estimated that one in five persons will get cancer in a lifetime and one in ten 10 persons will die from the disease [3].

Breast and cervical cancers remain the most common female cancers and the leading cause of cancer deaths in Ghana. In 2018, 22,823 cancer cases were recorded in Ghana with breast and cervical cancers accounting for 20.4% and 13.5%, respectively [4]. The number of

new cancer cases in Ghana increased to 24,009 in 2020. Breast cancer remained the most commonly reported while lung cancer overtook cervical cancer as the second highest [5].

Apart from the huge economic cost that cancer treatment poses to patients and their families, they also bear high psychosocial burdens ~~or intangible costs~~. While considerable work has been done on the increasing incidence and prevalence of cancer diseases in Ghana, there is limited literature on the experiences of cancer patients in the Ghanaian context on the burden and challenges they face in seeking treatment. Also, studies on the cancer burden on patients have focused on the economic aspect to the neglect of the ~~intangible costs which constitute the~~ psychosocial burden patients bear in seeking treatment for their illness [6]. This paper examines the psychosocial experiences of breast and cervical cancer patients who sought treatment in three hospitals in Ghana. The goal of this paper is to shed light on the ~~intangible costs or~~ burdens that breast and cervical cancer patients bear that ~~cannot be easily quantified in economic terms.~~

2.0 Methodology

Breast and cervical cancer patients were purposively selected from two public hospitals and one private hospital dedicated to cancer care in Ghana namely Korle-Bu and Komfo Anokye Teaching hospitals, and Sweden Ghana Medical Centre. The Korle-Bu Teaching Hospital (KBTH), situated in Accra, Ghana’s capital city, is the leading national referral centre in Ghana and the third largest hospital in Africa [7]. It is located in the southernmost part of Ghana. Komfo Anokye Teaching Hospital (KATH) is located in Kumasi, the regional capital of the Ashanti Region, in the middle part of Ghana. KATH is the second-largest Teaching Hospital in Ghana. KATH serves as a referral centre for 12 out of the 16 administrative regions in Ghana [8], which are in the middle to the northernmost part of the country. The

Sweden Ghana Medical Centre (SGMC), also located in Accra, is a leading private centre for cancer care in Ghana.

Patients who were seeking treatment at these hospitals were purposively selected using criterion sampling and based on certain predetermined inclusion criteria. Patients should be eighteen years and above; clinically diagnosed of breast or cervical cancer and undergoing biomedical treatment. Patients who were eligible to participate in the study were approached, with the help of oncology doctors and nurses, and those who gave informed consent were recruited as study participants.

Two hundred and ninety-eight (298) cancer patients took part in the study; 217 were breast cancer patients, and the remaining 81 were cervical cancer patients. Out of the 217 breast cancer patients, 115, 71 and 31 were from the Korle-Bu Teaching Hospital, Komfo Anokye Teaching Hospital, and the Sweden Ghana Medical Centre respectively. Out of the 81 cervical cancer patients, 46 sought treatment at Korle-Bu Teaching Hospital, while the remaining 35 were at Komfo Anokye Teaching Hospital. No cervical cancer patients from the Sweden Ghana Medical Centre were included in the study because none were receiving care in that facility at the time of data collection. Forty-five (45) respondents were followed up for qualitative interviews after they had participated in the initial survey. Twenty (20) of these 45 patients were from the Korle Bu teaching Hospital, fifteen (15) were from the Komfo Anokye Teaching Hospital, and ten (10) were from the Sweden Ghana Medical Centre. Informed by previous research [9, 10], qualitative interviews for patients at the various hospitals were halted once saturation points were reached and new interviews did not provide new insights into the phenomena being investigated.

A convergent mixed method approach was used for the study where quantitative and qualitative data were collected and analysed simultaneously [11]. A questionnaire and an

interview guide were used to gather quantitative and qualitative data, respectively. Patients responded to a series of yes or no questions on intangible cost such as pain, anxiety, stigma, physical and social restrictions. Frequency tables yielded the percentages of patients who experienced the various dimensions of intangible cost. Additionally, a multivariate logistic regression analysis was conducted (using patients' socio-demographic characteristics as independent variables and selected measures of intangible cost as dependent variables) to explore which patients were more prone to experience intangible cost. The quantitative data were analysed using Microsoft Excel 360 and STATA 16.1 while the qualitative data were transcribed verbatim and analysed using thematic content analysis (see for instance [9, 10])

3.0 Findings and Results

Patients reported psychological and emotional trauma resulting from worry, anxiety, fear, and financial-related stress. Patients also reported that they battled with physical and social restrictions and perceived stigma.

3.1 Anxiety and financial-related stress

Almost all the patients in the study had anxieties about their general health and the outcome of their treatment. They battled with financial-related stress as they worried about getting money for their treatment. Patients also worried about how to meet the needs of their dependents. Most patients worried about the burden they posed to their family members and loved ones. The quantitative analysis stress (Table 1) showed that 91.4% and 71.0% of breast cancer patients in public and private hospitals, respectively, reported anxiety. Eighty-six percent (86%) of cervical cancer patients also reported being worried because of their illness. About 90.9% and 19.4% of breast cancer patients in the public and private hospitals in the study reported financial related stress while 92.6% of cervical cancer patients experienced financial related. Majority of breast cancer patients who sought treatment at the private

hospital, mostly teachers, received comprehensive health insurance from the Ghana National Association of Teachers' (GNAT) cancer fund which reduced their financial stress.

Table 1: Intangible Cost of Breast and Cervical Cancer Treatment in Public and Private Hospitals

	Public Hospital		Private Hospital
**Statements**	Breast	Cervical	Breast
**Anxiety and fear**			
I often get worried because of my illness	91.4	86.4	71.0
I am sometimes afraid that I will die	87.6	82.7	77.4
I am afraid that I may lose my husband (based on married respondents only)	80.1	85.2	64.5
I experience financial related stress	90.9	92.6	19.4
There is stress on other members of my household, and I am worried about it	83.3	91.4	48.4
**Social and Physical Restriction**			
I am unable to attend social events as I used to	81.7	82.7	74.2
I must rest more, I feel restricted	88.2	85.2	83.9
I must change the food I eat; I feel restricted	70.4	72.8	83.9
I go through much physical and emotional pain	80.7	93.8	83.9
**Stigma and psychosocial distress**			
I struggle with perceived stigma	42.5	60.5	48.4
I must try hard to hide my illness from people	80.1	77.8	77.4

~~Source: Authors' data~~

With regards to worry, a 45-year-old cervical cancer patient stated:

> *"I get very worried; nobody likes to be sick. Your life gets disrupted. I am worried that I cannot care for my family the way I want to. I worry about the stress my husband is going through."*

A 47-year-old breast cancer patient, also noted:

> *"I worry about who will take care of my children when I am no more. My husband passed away during my treatment."*

Regarding financial related stress, a 55-year-old cervical cancer patient lamented:

> *"I get very anxious anytime my appointment date is getting close. How to get money for treatment is always a challenge. When you see that I am sitting quietly, it means I am in deep thought. Worry alone can even kill you. The cost of treatment is too huge for us."*

A 44-year-old breast cancer patient also bemoaned:

> *"It is the financial stress that is killing us…you see; I worry so much about how to get money for the treatment. When you worry, you can't eat, you can't sleep! If you know that there is money for treatment, you don't worry, you just pray that the appointment date will come quickly so that you can complete your treatment and be healed…When you start treatment with someone at the same time and they have money, they will complete their treatment very fast, and you will still be here struggling."*

Even though most of the patients knew that worry was not good for their health, they could simply not avoid it because of the financial burden they faced and the consequences of not being able to raise money for treatment. Even though relatives and oncology staff encouraged patients to desist from worrying, it was not enough to allay the anxieties as their challenges persisted.

A multivariate logistic regression (Table 2) revealed a statistically significant association between type of cancer, patient's highest level of education and worrying among patients due to their illness. Cervical cancer patients had significantly lower odds [OR = 0.29 (95% CI; 0.11, 0.65), p=0.003] of worrying due to their illness compared with patients with breast cancer. Respondents with secondary [OR = 0.19 (95% CI; 0.04, 0.99), p=0.048] and tertiary [OR = 0.05 (95% CI; 0.01, 0.29), p=0.001] levels of education were less likely to worry about their illness compared with those with no formal education. Patients who were highly educated and could read about cancer experienced less anxiety about dying from their illness compared to patients who could not read and had little information about cancer.

Table 2: Determinants of Worrying among Patients due to Illness Among Respondents

**Variable**	**OR [95% CI]**	**P-value**
Occupation		
Waged worker	1.00	
Self-Employed	0.73 [0.25, 2.13]	0.566
Unemployed	0.62 [0.06, 6.67]	0.691
Retired	0.57 [0.18, 1.81]	0.337

*Monthly Income (GHC/USD)		
Less than GHC 500 ($89.2)	1.00	
GHC 500-999 ($89.2 -178.4)	0.71 [0.25, 2.03]	0.523
GHC 1000-1999 ($178.6 -356.9)	1.43 [0.45, 4.48]	0.543
GHC 2000-2999 ($357.1 -535.5)	0.80 [0.23, 2.82]	0.727
GHC 3000-3999 ($535.7-714.1)	0.90 [0.13, 6.24]	0.913
GHC 4000+ ($714.3)	0.49 [0.07, 3.29]	0.459
Stage of cancer on diagnosis		
Early stage	1.00	
Middle stage	1.25 [0.55, 2.82]	0.591
Advanced stage	1.62 [0.16, 6.17]	0.679
Residential Status		
Rural	1.00	
Urban	0.86 [0.35, 2.13]	0.745
Type of Hospital		
Public	1.00	
Private	3.62 [0.68, 9.21]	0.131
Type of cancer		
Breast cancer	1.00	
Cervical cancer	0.27 [0.11, 0.65]	**0.003**
Highest level of education		
No formal education	1,00	
Primary	0.29 [0.05, 1.61]	0.157
Secondary	0.19 [0.04, 0.99]	**0.048**
Tertiary	0.05 [0.01, 0.29]	**0.001**

OR = Odds Ratio, CI = Confidence Interval.

*(Inter-Bank Exchange Rate-Month Average, GHC5.6 =1 USD, June 2020 (when data was collected)

Source: Authors' data, 2022

3.2 *Fear of death, loss of spouses and care for young dependents*

Patients also battled with fear of the unknown as they were not sure of the outcome of their treatment. This was true especially for those who had financial challenges as they were afraid that they might die if they did not get money to complete their treatment on time. Other patients had fears about losing their spouses to divorce or separation due to the high financial burden on their spouses, stemming from treating their cancer. Some patients reported that their spouses gave up on them or threatened to do so if their illness worsened.

Most (87.6% and 77.4%) of breast cancer patients in the public and private hospitals studied, respectively, and 82.7% of the cervical cancer patients studied had fears that they would die because of their illness. The majority, 80.1% of breast cancer patients in the public hospitals compared to 64.5% in the private hospital feared losing their husbands to divorce or separation because of their illness. Similarly, 85.2% of cervical cancer patients had fears of losing their husbands to divorce or separation due to their illness (Table 1). A 48-year-old cervical cancer patient said her greatest fear was who would take care of her children should she die. Her fear was not of death but the upkeep and well-being of her young dependants. She explained:

> *"I am not necessarily afraid of death; my fears are about who will take care of my children when I am no more. It is my biggest fear."*

A 51-year-old breast cancer patient also stated:

> *"I am sometimes afraid that I may die because you see people who die because of their illness."*

A 34-year-old cervical cancer patient recalled:

> *"A lot of the people I started the treatment with have died. Sometimes it really gets to you, and you get scared that you might not live long. If you don't have strong support from your family, you will die. Even the side effect of the treatment alone can kill you."*

A 41-year-old breast cancer patient also lamented:

> *"I am afraid that I may lose my husband because his attitude has changed due to the huge financial burden."*

The fear of death among the respondents was significantly associated with the stage of cancer, type of health facility the patient received care at, and the highest level of education attained by the patient. Patients who were at the advanced stage of cancer were more likely [OR = 3.82 (95% CI; 1.23, 6.55), p=0.032] to experience fear of death compared with those patients at their early stage of cancer diagnosis. Patients who received care at the public healthcare facilities were more likely [OR = 5.19 (95% CI; 1.80, 15.00), p=0.002] to

experience fear of death relative to those patients who received care at the private healthcare facility. Patients with secondary [OR = 0.18 (95% CI; 0.07, 0.46), p<0.001] and tertiary [OR = 0.08 (95% CI; 0.03, 0.24), p<0.001] level education were less likely to experience fear of death due to their illness compared with those patients with no formal education (Table 3).

Patients who were diagnosed with advanced-stage cancer and had lower chances of cure reported fear of death as a major psychological burden compared with patients who were diagnosed with early-stage cancer and had higher chances of cure. Also, patients who sought treatment at the private hospital reported that they received prompt and better care, easy access to oncologists, shorter waiting periods at the hospital compared with patients who sought treatment at the public hospitals. With the high quality of care patients received at the private hospital, they hoped for speedy completion of the treatment regimen and resultant cure for their ailment, which reduced the fear of possible death due to cancer. Also, patients with higher education who read a lot about their illness and treatment procedures had their fear of death due to cancer allayed compared with patients with little or no formal education and could not read.

Table 3: Determinants of Fear of Death among Patients due to Illness Among Respondents

Variable	OR [95% CI]	P-value
Occupation		
Waged worker	1.00	
Self-Employed	0.75 [0.32, 1.76]	0.515
Unemployed	1.11 [0.19, 6.38]	0.904
Retired	0.79 [0.31, 2.00]	0.615
*Monthly Income (GHC/USD)		
Less than GHC 500 ($89.2)	1,00	
GHC 500-999 ($89.2 -178.4)	1.06 [0.51, 2.18]	0.883
GHC 1000-1999 ($178.6 -356.9)	1.15 [0.53, 2.46]	0.728
GHC 2000-2999 ($357.1 -535.5)	1.12 [0.44, 2.87]	0.807
GHC 3000-3999 ($535.7-714.1)	2.03 [0.46, 8.95]	0.352
GHC 4000+ ($714.3)	0.48 [0.10, 2.29]	0.356
Stage of cancer on diagnosis		

Early stage	1,00	
Middle stage	1.24 [0.68, 2.27]	0.489
Advanced stage	3.82 [1.23, 6.55]	**0.032**
Residential Status		
Rural	1.00	
Urban	1.27 [0.69, 2.32]	0.439
Type of Hospital		
Public	5.19 [1.80, 15.00]	**0.002**
Private	1.00	
Type of cancer		
Breast cancer	1.00	
Cervical cancer	0.60 [0.31, 1.17]	0.133
Highest level of education		
No formal education	1,00	
Primary	0.75 [0.29, 1.95]	0.558
Secondary	0.18 [0.07, 0.46]	< **0.001**
Tertiary	0.08 [0.03, 0.24]	< **0.001**

*(Inter-Bank Exchange Rate-Month Average, GHC5.6 =1 USD, June 2020 (when data was collected)

Source: Authors' data, 2022

3.3 *Emotional and physical pain*

Most (80.7%) of the breast cancer patients studied in the public hospitals and 83.9% of respondents from the private hospital reported that they went through physical and emotional pain. Likewise, 93.8% of cervical cancer patients reported that they experienced much pain because of their illness (see Table 1). A 34-year-old cervical cancer patient reported:

> *"Sometimes you get very weak and have pains all over your body especially after chemotherapy. You are unable to rise from your bed for about 3 days to eat or do anything. Sometimes too you vomit a lot."*

A 48-year-old breast cancer patients also stated:

> *"I experience a lot of pain from the surgical wounds and the area around my armpit. My arms get swollen and very painful sometimes."*

With reference to emotional pain, a 57-year-old breast cancer patient also revealed:

> *"If a doctor prescribes medication for you which you know will make you well, but you don't have money to buy and you know your illness is getting worse, what will you do? Won't you shed tears?"*

A 47-year-old cervical cancer patient also added:

> *"I cry a lot because of this illness and the associated cost. The nurses know me as the crying patient. When they mention the amount of money I must pay, I just burst into tears. I do not know where this illness came from. It has brought so much financial distress to my family. I have become a beggar, always asking for money for my treatment."*

3.4 *Dietary, physical, and social restrictions*

The onset of cancer disrupted the physical and social lives of patients. Apart from the physical discomfort they were experiencing due to the illness, their social activities were halted or slowed. Patients spent several hours at the hospital seeking treatment, they also battled with low energy, physical restrictions, and side effects of treatment. As a result, their participation in family gatherings, church and other religious activities, funerals and parties was severely limited.

About 81.7% of the breast cancer patients studied in the public hospitals, compared to 74.2% of those studied in the private hospital, were unable to attend social events as they used to because of their illness. The majority (82.7%) of cervical cancer patients were also unable to attend social events as they used to. About 88.2% and 83.9% of breast cancer patients studied in the public and private hospitals felt physically restricted because of the need to rest more. Eighty-five percent (85.2%) of cervical cancer patients studied also experienced physical restrictions (see Table 1).

A 52-year-old cervical cancer patient noted:

> *"The side effects of the treatment were such that I changed completely. My whole body darkened, and I lost my hair. Because of that I hardly go out. I do not remember the last time I attended a funeral or some social event."*

A 41-year-old breast cancer patient stated that she wanted to avoid interrogations from people regarding her illness. As a result, she avoided social gatherings. She highlighted that even though she tried very hard to cover the side effects of treatment by wearing wigs, nail polish and make up, it was hard to cover her swollen arms. She was afraid someone might notice it and start asking questions. She explained:

> *"I have stopped attending social events. I don't want anyone to spot the side effects of treatment and start asking questions… besides, where is the energy and money for social activities?"*

This patient also complained about her low energy levels and bad financial situation which did not promote social participation.

A 43-year-old breast cancer patient stated,

> *"As part of the side effects of chemotherapy, my right arm got swollen for a long time. I stopped attending social events because people noticed it and started asking questions."*

Furthermore, about 70.4% of the breast cancer patients studied in public hospitals and 83.9% of those studied in the private hospital felt restricted because of dietary changes. Seventy-three percent (73.0%) of the cervical cancer patients studied also reported feeling restricted due to dietary changes (see Table 1). Patients were put on dietary restrictions to help with their recovery and general well-being. Regarding dietary restrictions, a 53-year-old cervical cancer patient indicated:

> *"I have been told to stay away from red meat, oily food, and milk. The doctors advise that I should eat more fruits and vegetables, but these are more expensive."*

3.5 *Perceived Stigma and psychosocial distress*

Stigma and confidentiality regarding cancer played out very evidently among some patients. About 42.5% and 48.4% of breast cancer patients studied in the public and private hospitals,

respectively, and 60.5% of the cervical cancer patients studied, reported that they struggled with perceived stigma. As a result, 80.1% and 77.4% of the respondents studied in the public and private hospitals, respectively, hid their cancer diagnosis from people. Similarly, 77.8% of the cervical cancer patients studied also kept their diagnosis a secret from the public (see Table 1).

Most patients indicated that even though people may know in some cases that they were not well, they did not know that it was cancer. Their cancer diagnosis was shared with only close family members and friends who were involved in their treatment process. This included people who accompanied them to the hospital or supported them financially.

A 56-year-old cervical cancer patient explained:

> *"My neighbours know that I am sick, but they are not aware that it is cancer. I told them the illness is in my stomach."*

A 54-year-old breast cancer patient who was a head teacher concealed her diagnosis from her colleague teachers. She told her staff that she was going for a meeting whenever she had to go for chemotherapy or radiotherapy sessions. When asked why she chose to be silent about her cancer diagnosis, she indicated:

> *"People gossip a lot; I don't want anybody to know my business. And when you have cancer, everybody thinks you are about to die but cancer can be treated. Do I look like someone who is about to die?"*

Fortunately, this patient was very strong and healthy. She was well to do financially, and she also received financial support from the Ghana National Association of Teachers (GNAT) cancer fund. Also, because she was the head teacher, she did not need permission from anyone at work before going to the hospital for her treatment. She could afford to eat well to maintain her health.

While some patients came to the hospital with their spouses and other family members, a 38-year-old breast cancer patient concealed her diagnosis from her husband and

relatives. Her reason was that if her husband got to know about her condition, her mother in-law would automatically be aware and that would mean a broadcast to her entire community. So, she chose to keep the details about her condition to herself. She explained:

> *"Even though my husband knows that there is a problem with my breast, he doesn't know that it is cancer. He is aware that I come to the hospital, but he doesn't know I am doing chemotherapy. I always wear wig in the house to cover my hair loss. I have a very troublesome mother in-law."*

Being a teacher, this patient had support from the GNAT cancer fund, and her husband supported her transportation to the hospital. However, probably because she concealed her disease from her close family members, she lacked the emotional support that most cancer patients had from close relatives.

The regression analysis (Table 4) showed that stigmatization due to illness was significantly associated with patient's occupation and type of cancer. Respondents who were self-employed were more likely [OR = 2.50 (95% CI; 1.10, 5.70), p=0.030] to be stigmatized due to their illness when compared with waged workers. Cervical cancer patients compared with breast cancer patients were more likely [OR = 1.95, (95% CI; 1.04, 3.65), p=0.036] to be stigmatized due to their illness.

Table 4:Determinants of Stigmatization due to Illness Among Respondents

Variable	OR [95% CI]	P-value
Occupation		
Waged worker	1.00	
Self-Employed	2.50 [1.10, 5.70]	**0.030**
Unemployed	2.54 [0.53, 7.28]	0.245
Retired	1.07 [0.42, 2.75]	0.889
*Monthly Income (GHC/USD)		
Less than GHC 500 ($89.2)	1.00	
GHC 500-999 ($89.2 -178.4)	0.62 [0.31, 1.22]	0.165
GHC 1000-1999 ($178.6 -356.9)	0.79 [0.39, 1.60]	0.508
GHC 2000-2999 ($357.1 -535.5)	1.00 [0.42, 2.40]	0.995
GHC 3000-3999 ($535.7-714.1)	2.92 [0.64, 13.35]	0.167
GHC 4000+ ($714.3)	0.42 [0.09, 2.09]	0.291
Stage of cancer on diagnosis		

Early stage	1.00	
Middle stage	0.65 [0.37, 1.16]	0.148
Advanced stage	1.03 [0.21, 4.92]	0.974
Residential Status		
Rural	1.00	
Urban	0.93 [0.53, 1.64]	0.803
Type of Hospital		
Public	1.00	
Private	1.81 [0.69, 4.73]	0.226
Type of cancer		
Breast cancer	1.00	
Cervical cancer	1.95 [1.04, 3.65]	**0.036**
Highest level of education		
No formal education	1.00	
Primary	1.12 [0.49, 2.54]	0.794
Secondary	0.68 [0.30, 1.53]	0.354
Tertiary	0.81 [0.30, 2.16]	0.670

*(Inter-Bank Exchange Rate-Month Average, GHC5.6 =1 USD, June 2020 (when data was collected)

Source: Authors' data, 2022

4.0 Discussion

Breast and cervical cancer patients in the study experienced high intangible cost of treatment, including anxiety, fear, pain, stress, physical and social restrictions. Patients with low educational level (no formal education and primary education), who were retired or unemployed, who earned less monthly income and had larger household size, suffered more intangible cost compared to patients with high educational level (secondary and tertiary education) who were gainfully employed, earned higher income, and had smaller household size. This finding supports earlier studies where Cvetković and Nenadović [12] documented depression among 84 breast cancer patients undergoing therapy at the Clinical Centre of Niš in Serbia. Adanu [13] recorded low and moderate intangible burden among 74 breast cancer patients he studied at the Korle-Bu Teaching Hospital in Ghana. Gyau [14] however, found a high intangible burden among 56 breast cancer patients she studied at the Korle-Bu Teaching

Hospital in Ghana. Kugbey, Oppong Asante and Meyer-Weitz [15] reported depression among 205 breast cancer patients who sought treatment at the Korle-Bu Teaching Hospital in Ghana. Alexander et al. [16] found social embarrassment among 378 breast cancer patients seeking treatment at St. John's Research Institute in India. Kugbey, Meyer-Weitz and Oppong Asante [17] highlight that negative psychosocial burden affects the quality of life of cancer patients. Similar experiences were recorded in China where high anxiety was found to have adversely affected the quality of life of cancer patients [18].

Our study participants felt the need to keep their diagnosis secret to most people, except a few close family members; principally, persons who accompany them for hospital visits and those who provide financial support to them, for purposes of avoiding stigma. This finding is corroborated by previous studies which have reported social stigma of certain diseases in Ghana including HIV/AIDS, epilepsy, and leprosy [9, 10, 19]. Psychosocial burdens such as social stigma, isolation, and divorce have also been reported among people living with Buruli ulcer disease in Ghana [20, 21]. Stigmatization has the tendency to interfere with treatment, economic wellbeing, and quality of life of patients. For instance, other previous researchers have shown that HIV/AIDS patients may not adhere to ART medications or engage in economic activities due to fear of stigma [9, 22]. Some Buruli ulcer patients in Ghana chose to seek alternative treatment such as herbal medicine instead of reporting for free treatment at hospitals due to stigma [20]. Owusu and Adamba [20] also reported that the career aspirations of Buruli ulcer patients were limited due to the stigma associated with the condition. Like Buruli ulcer sufferers, some breast and cervical cancer patients in our study chose to forgo the emotional and financial support from other people such as neighbors due to the need to hide their illness for fear of stigma [20, 21].

Cervical cancer patients in our study were more likely to experience stigma than breast cancer patients. The increased stigma attached to cervical cancer can be explained by

the known risk factors such as HPV infections (particularly HPV types 16, 18, 31, 33, and 45), multiple sexual partners and young age at first coitus [23]. For instance, Kashyap, et al [23] found that prominent among cervical cancer risk factors in India were history of sexually transmitted diseases and genital warts, increased number of husband's sexual partners and early age of marriage. The fact that cervical cancer is associated with a sexually promiscuous lifestyle increases the stigma attached to it. For example, Kashyap, et al [23] emphasize that women in rural India ignore the signs of cervical cancer because of shyness.

Breast and cervical cancer patients in our study also experienced social and physical restrictions resulting from hospitalisation, body weakness and pain emanating from treatment procedures such as surgery and chemotherapy. Side effects of treatment such as weight loss, darkened palms and feet, and hair loss were visible to others and raised suspicions about their health status. This deterred our respondents from attending social gathering, a phenomenon which resonates with Buruli ulcer sufferers who isolate themselves because of their illness [20, 21]. Like Buruli ulcer sufferers, some breast and cervical cancer patients had fears of losing their spouses to separation or divorce or actually lost them due to their illness. For one of our respondents to work on, and succeed in hiding her cancer from her husband attests to the strong stigma and psychosocial burden of the disease on patients, within the Ghanaian cultural setting. This situation exacerbates feelings of isolation, loneliness, and self-pity among patients.

Our finding that our respondents suffering from cervical cancer had significantly lower tendency to worry due to their illness compared with the respondents with breast cancer is very interesting. This may be because both internationally [3], and also in Ghana [4, 5], there are more breast cancer cases than cervical cancer cases. Furthermore, there are more deaths from breast cancer, compared to cervical cancer, both internationally, and in sub-Saharan Africa [3]. These may have influenced why breast cancer is more known and talked

about in Ghana, compared to cervical cancer, probably influencing our cervical cancer patients worrying less about their disease than those with breast cancer.

Interestingly, Huang et al.'s [24] study on the quality of life (QOL) of breast and cervical cancer patients in eight major hospitals in Northern, Central and Southern Taiwan, concluded that their two groups of respondents had similar QOL; the breast cancer survivors they studied had several multiple factors influencing their QOL while their cervical cancer survivors had only one factor: household income which affected their QOL. Importantly, while the psychosocial burden of cancer treatment is challenging to quantify, it is a vital aspect of breast and cervical cancer burden and must be highlighted.

5.0 Conclusion

We studied the psychosocial burden of breast and cervical cancer treatment for patients in three leading cancer hospitals in Ghana. Our study found that the patients studied experienced psychosocial burdens such as anxiety and fear, physical and social restrictions, stigma, and stress from their illness. Also, respondents had anxieties about their treatment outcome, while some feared that they might die or lose their spouses to divorce or separation due to the stress from their illness. The majority of patients studied also experienced financial-related stress due to the high cost of treatment, especially those who had very little financial resources of their own or support from others. Additionally, most of our respondents were unable to attend social events like they used to do before the onset of cancer. The majority of the respondents also reported going through lots of pain and felt the need to get adequate rest. In sum, we conclude that the breast and cervical cancer treatment costs for the patients we studied are enormous, go beyond financial burden, and disrupt their lives and that of their households.

There is a need for psychological support such as free and routine counselling services for the cancer patients we studied and their caregivers. Other studies in Ghana have

underscored such need to help persons dealing with ailments such as HIV/AIDS better cope with their illness [9, 17]. This is crucial in alleviating the fears and anxieties of patients. Social support from friends and family is also critical to the well-being of our respondents. There is also need for increased funding from the National Health Insurance for breast and cervical cancer treatment in Ghana. Educational campaigns on mainstream and social media have contributed to increased awareness on breast and cervical cancers in Ghana. The activities of organizations such as Breast Care International and Pink Africa have been critical in increasing public education on cancers in Ghana. There is, therefore, the need for increased educational campaigns to demystify the stigma surrounding cancers in Ghana.

Acknowledgement

We thank the patients who shared their experiences with us. We are also grateful to the oncology doctors, nurses and other health personnel who helped in making the fieldwork successful.

Author contributions

Patience Nana Amoa Gyamenah: Conceptualisation, Methodology, Validation, Formal Analysis, Investigation, Resources, Data Curation, Writing—Original Draft, Writing---Review & Editing, Visualisation, Project Administration.

Adobea Yaa Owusu: Conceptualisation, Methodology, Validation, Formal Analysis, Investigation, Resources, Data Curation, Writing---Review & Editing, Visualisation, Supervision, Project Administration.

Joseph Ransford Oppong: Conceptualisation, Methodology, Validation, Resources, Writing---Review & Editing, Visualisation, Supervision.

Kingsley E. Amegah: Methodology, Validation, Formal Analysis, Resources, Data Curation, Writing—Original Draft, Visualisation.

Edward Nketiah-Amponsah: Conceptualisation, Methodology, Validation, Investigation, Resources, Writing---Review & Editing, Visualisation, Supervision.

Data availability: The data for this paper are available upon reasonable request from Dr. Patience Nana Amoa Gyamenah, upon a formal request. There are ethical and confidential concerns about breast cancer within the Ghanaian cultural setting. The patients were promised that their responses will not be shared with the public, unless it becomes necessary to share it with persons who may request to use it privately—or to Editors of publishing outlets, when some of them showed concerns during the interviews about keeping the data confidential.

Questions on the data **can be directed at Dr.** Patience Nana Amoa Gyamenah **(**Institute of Statistical, Social and Economic Research. University of Ghana, Legon. Accra, Ghana; email: patg2014@gmail.com).

Ethics approval

Ethical clearance was obtained from the Ethics Committee for Humanities of the University of Ghana (Ref. No ECH011/19-20), the Korle-Bu Teaching Hospital Ethical Review Board (Ref. No KBTH/MD/G8/20), and the Komfo Anokye Teaching Hospital Review Board (Ref. No KATH-IRB/AP/023/20). Informed written consent was obtained from all study participants before interviews were conducted. Permission was also obtained from research participants to record interviews for the purposes of data analysis and for writing academic-related reports.

Funding: This research received no funding outside the resources of the authors.

Competing interests: None declared.

References

1. Beaglehole, R., et al., Priority actions for the non-communicable disease crisis. Lancet, 2011. **377**(9775): p. 1438-47.
2. Sung, H., et al., Global cancer statistics 2020: GLOBOCAN estimates of incidence and mortality worldwide for 36 cancers in 185 countries. CA: a cancer journal for clinicians, 2021. **71**(3): p. 209-249.
3. Ferlay, J., et al., Cancer statistics for the year 2020: An overview. International Journal of Cancer, 2021.
4. IARC, New Ghana Cancer Data. International Agency for Research on Cancer, 2018.
5. IARC, Ghana cancer statistics. International Agency for Research on Cancer, 2020.
6. Zelle, S.G., et al., *Costs, effects and cost‐ effectiveness of breast cancer control in Ghana.* Tropical Medicine & International Health, 2012. **17**(8): p. 1031-1043.
7. KBTH, K.-B.T.H. Abou us-Brief History [Internet]. 2019. Available from: https://kbth.gov.gh/brief-history/.
8. KATH, K.A.T.H. Our History, Mission and Vision [Internet]. 2019. Available from: http://www.kathhsp.org/?page_id=370.
9. Owusu, A.Y. Social contexts of living with HIV/AIDS in the Eastern Region of Ghana. Istanbul University Journal of Sociology, 2019. **39**(2): p. 425-454.
10. Owusu, A.Y. A gendered analysis of living with HIV/AIDS in the Eastern Region of Ghana. BMC Public Health, 2020. **20**(1): p. 1-15.

11. Guetterman, T. C., Fetters, M. D., & Creswell, J. W. (2015). Integrating quantitative and qualitative results in health science mixed methods research through joint displays. *The Annals of Family Medicine, 13*(6), 554-561.

12. Cvetković, J. and M. Nenadović, Depression in breast cancer patients. Psychiatry research, 2016. **240**: p. 343-347.

13. Adanu, K.K., Household Treatment Cost of Breast Cancer Disease and Cost Coping Strategy of Women, Presenting at the Korle Bu Teaching Hospital. 2019, University of Ghana.

14. Gyau, D.P., Economic Burden of Breast Cancer in Women: A study at the Korle-Bu teaching hospital. 2016, University of Ghana.

15. Kugbey, N., K. Oppong Asante, and A. Meyer-Weitz, Depression, anxiety and quality of life among women living with breast cancer in Ghana: mediating roles of social support and religiosity. Supportive Care in Cancer, 2020. **28**(6): p. 2581-2588.

16. Alexander, A., et al., The impact of breast cancer on the patient and the family in Indian perspective. Indian journal of palliative care, 2019. **25**(1): p. 66.

17. Kugbey, N., A. Meyer-Weitz, and K. Oppong Asante, Mental adjustment to cancer and quality of life among women living with breast cancer in Ghana. The International Journal of Psychiatry in Medicine, 2019. **54**(3): p. 217-230.

18. Owusu, A.Y. Experiences of new diagnoses among HIV-positive persons: implications for public health. BMC public health, 2022. **22**(1): p. 1-17.

19. Gong, Y., Yan, Y., Yang, R., et al. Factors influencing death anxiety among Chinese patients with cancer: a cross-sectional study. BMJ Open 2022; 12:e064104. doi:10.1136/bmjopen-2022-064104

20. Owusu, A.Y. and Adamba, C. The socioeconomic burden of Buruli ulcer disease in the GA West District of Ghana. Ghana Journal of Development Studies, 2012. 9(1): p. 5-20.

21. Owusu, A.Y. and C. Adamba, Household perceptions, treatment-seeking behaviour and health outcomes for buruli ulcer disease in a peri-urban district in Ghana. 2012.

22. Poku, R.A., et al., Antiretroviral therapy maintenance among HIV-positive women in Ghana: the influence of poverty. AIDS care, 2020. **32**(6): p. 779-784.

23. Kashyap, N., Krishnan, N., Kaur, S., & Ghai, S. (2019). Risk factors of cervical cancer: a case-control study. *Asia-Pacific journal of oncology nursing*, *6*(3), 308-314.

24. Huang, H. Y., Tsai, W. C., Chou, W. Y., Hung, Y. C., Liu, L. C., Huang, K. F., ... & Kung, P. T. (2017). Quality of life of breast and cervical cancer survivors. *BMC women's health*, *17*(1), 1-12.
